# Immunomodulatory Effects of *Lactobacillus brevis* NES-428 in a Hyperthyroidism Mouse Model: Potential Applications for Graves’ Disease

**DOI:** 10.3390/nu17182967

**Published:** 2025-09-16

**Authors:** Min-Gyu Lee, Dong-Hyun Lee, Suzie Kang, Jongho Koh, Cheol-Won Yun

**Affiliations:** 1 School of Life Sciences and Biotechnology, Korea University, Anam-dong, Sungbuk-gu, Seoul 02841, Republic of Korea; 2 Department of Convergence Industry, Seoul Venture University, Seoul 06097, Republic of Korea; 3 EsgelBio Co., Anam-dong, Sungbuk-gu, Seoul 02841, Republic of Korea

**Keywords:** Graves’ disease, probiotics, *Lactobacillus brevis*, hyperthyroidism, immunomodulation

## Abstract

**Background:** Safe, microbiome-based interventions for autoimmune hyperthyroidism are lacking. We isolated the lactic acid bacterium NES-428 from kimchi and previously demonstrated that it shares 99% 16S-rRNA identity with *Lactobacillus brevis* reference strains, confirming NES-428 as a novel strain. Here we evaluated its immunomodulatory and anti-thyroid activity in cellular and murine models. **Methods:** Jurkat T cells (5 × 10^6^) were incubated with heat-killed NES-428 for 24 h and subsequently stimulated with phorbol 12-myristate 13-acetate/ionomycin (50 ng mL^−1^/1 µg mL^−1^) for 6 h; cytokine transcripts were quantified by qRT-PCR. Hyperthyroidism was induced in female BALB/c mice by three intramuscular injections of adenovirus-encoding human TSH receptor (Ad-TSHR). Mice received a daily oral dose of NES-428 (1 × 10^9^ CFU) for 15 weeks. Serum thyroxine (T_4_) levels, splenocyte cytokine secretion, and thyroid histopathology were assessed. Statistical analyses employed one-way ANOVA with Tukey post hoc or log-rank tests (α = 0.05). **Results:** NES-428 pre-conditioning of Jurkat cells significantly down-regulated *IL-2* and *IFN-γ* transcripts (−48% and –43%, respectively; *p* < 0.01) compared with stimulated controls while modestly increasing *IL-4* (+26%). In Ad-TSHR mice, daily NES-428 reduced mean serum T_4_ from 11.2 ± 2.1 to 5.8 ± 1.4 µg dL^−1^ (*p* < 0.001), restored body weight gain, and normalized follicular architecture relative to untreated hyperthyroid animals. NES-428 supplementation also lowered splenocyte IFN-γ secretion by 58% and raised IL-4 by 41% (*p* < 0.05). **Conclusions:** The kimchi-derived strain NES-428 attenuates Th1-skewed cytokine responses and ameliorates experimental hyperthyroidism in vivo. These findings support further investigation of NES-428 as a probiotic candidate for immune modulation in Graves’ disease.

## 1. Introduction

Graves’ disease is an autoimmune disorder in which the immune system attacks the thyroid gland, causing aberrant production of thyroid-stimulating hormone receptor antibodies (TRAb) [1]. It leads to excessive synthesis and release of thyroid hormones (T3 and T4) independent of physiological regulation and causes symptoms such as weight loss, rapid or irregular heartbeat, nervousness, irritability, trouble sleeping, muscle weakness, sweating, and heat intolerance. The thyroid gland often becomes enlarged (goiter), and some people develop eye problems (Graves’ ophthalmopathy), such as bulging eyes, irritation, or double vision [2]. The exact cause is unknown, but it is more common in women and may be influenced by genetic and environmental factors [3]. The global prevalence of GD ranges from 0.5% to 2% of the population, with a higher incidence in women and individuals with a family history of autoimmune diseases [4].

The etiology of GD is multifactorial, involving complex interactions between genetic factors, environmental triggers, and dysregulated immune responses [5]. In the immune system, GD is characterized by a breakdown in self-tolerance, leading to the activation of autoreactive T and B cells that target the TSHR. This process involves a complex interplay of pro-inflammatory cytokines, including IL-2, IL-6, TNF-α, IL-12, IFN-γ, and IL-4, which contribute to the activation of immune cells and the perpetuation of the autoimmune response [6]. Specifically, Th1-mediated responses, characterized by the production of IFN-γ and IL-12, promote cell-mediated cytotoxicity and thyroid inflammation, while Th2-mediated responses, driven by IL-4, lead to the production of TRAb by B cells [7]. Furthermore, a reduction in the number and function of regulatory T cells (Tregs), which are critical for maintaining immune homeostasis, has been observed in GD patients. This imbalance exacerbates the autoimmune response and contributes to the chronic disease [8].

Current therapeutic strategies for GD primarily focus on alleviating symptoms and suppressing thyroid hormone production. These include antithyroid drugs, radioiodine ablation, and thyroidectomy [9]. However, antithyroid drugs can cause side effects such as agranulocytosis and liver damage, and they often result in discontinuation [10,11]. Therefore, it is required for alternative therapies that can target the underlying immune dysregulation in GD and prevent disease progression.

Probiotics confer a health benefit on the host when administered in adequate amounts and have emerged as promising candidates for immunomodulatory interventions [12]. Several studies have demonstrated that probiotics can modulate immune responses by influencing cytokine production, promoting the development of Tregs, and altering the composition of the gut microbiota [6]. *Lactobacillus* species have been shown to exert beneficial effects in various autoimmune diseases by restoring immune homeostasis and reducing inflammation [13]. Heat-killed probiotics have also been found to possess immunomodulatory properties and may offer advantages over live probiotics, such as improved stability and reduced risk of infection [14]. Given the immunomodulatory potential of probiotics and the limitations of current GD therapies, this study aims to evaluate the effects of *Lactobacillus brevis* NES-428, a strain isolated from kimchi, on thyroid function and immune responses in a hyperthyroidism mouse model induced by TSHR overexpression. The NES-428 species was selected because it can regulate the thyroid hormone level. Additionally, we evaluate the probiotic’s characteristics and safety of its use, studying aspects such as the gastric juice viability and bile tolerance of NES-428. Our findings may provide insights into the development of probiotic-based preventive or therapeutic strategies for autoimmune thyroid diseases like Graves’ disease.

## 2. Materials and Methods

### 2.1. Isolation of Lactic Acid Bacteria from Korean Kimchi

To selectively isolate lactic acid bacteria (LAB), both commercial and homemade Kimchi samples were utilized, sourced from various regions across Korea. Each sample was first diluted in a 1:4 ratio (*v*/*v*) with sterile phosphate-buffered saline (PBS), then homogenized to ensure consistent distribution of microbial content. The resulting homogenates were serially diluted with PBS to reduce microbial density to a cultivable range. From each dilution level, 100 µL aliquots were inoculated onto de Man, Rogosa, and Sharpe (MRS, Sigma-Aldrich, St. Louis, MO, USA) agar plates. Plates were incubated at 37 °C for 2 days under aerobic conditions for growing LAB colonies. After incubation, colonies were selected and subcultured in fresh MRS broth. Isolates were suspended in MRS broth with 20% (*v*/*v*) glycerol and stored at −80 °C for long-term cryopreservation. For the identification of a pure single colony of lactic acid bacteria (LAB), the 16S rRNA of sub-cultured colonies was amplified using the SapphireAmp^®^ Fast PCR Master Mix (Takara Bio, Kusatsu, Japan). PCR amplification was carried out with the primers 27F (5 μM) (5′-GAGTTGGATCCTGGCTCAG-3′) and 1492R (5 μM) (5′-AAGGAGGGGATCCAGCC-3′). The PCR reaction mixture consisted of 25 μL of PCR Master Mix (2X Premix), 0.5 μM of each primer (27F and 1492R), and distilled water (dH2O) to a final volume of 50 μL. The thermal cycling conditions were set according to the manufacturer’s protocol. Following PCR amplification, the products were purified using the QIAquick PCR Purification Kit (Qiagen, Hilden, Germany) and subsequently subjected to sequencing. Sequencing was performed using primers 27F, 337F, 518F, 785F, 518R, 783R, 805R, 907R, and 1492R, with their sequences listed in Table 1. The sequencing was carried out using an Applied Biosystems 3730XL DNA Analyzer at Bionics (Seoul, Republic of Korea). The obtained 16S rRNA sequences were analyzed by comparing them with closely related strains using the NCBI Basic Local Alignment Search Tool (BLAST). To confirm purity and species identity, the 16S rRNA gene was amplified and sequenced, and the obtained sequences were compared with reference databases. Only isolates showing homogeneous colony morphology and a single 16S rRNA sequence were considered pure cultures.

### 2.2. Acid Tolerance Assay

To evaluate acid tolerance, mid-log phase cultures of bacteria grown in MRS broth were washed twice with 0.75% saline and then incubated in simulated gastric juice (pH 2.5), prepared by dissolving 0.3% (*w*/*v*) pepsin (Sigma-Aldrich, St. Louis, MO, USA) in sterile saline and adjusting the pH with 5N HCl. The suspension was incubated at 37 °C, and viability was assessed at 0, 3, 6, 9, 12, and 24 h by serial dilution and plating on MRS agar [15]. Colony-forming units (CFU) were used to determine viability.

### 2.3. Hemolytic Activity Assay

Lactic acid bacteria pre-cultured in MRS broth were streaked onto blood agar plates (Kisan Biotech, Seoul, Korea) containing 5% (*w*/*v*) defibrinated sheep blood and incubated at 37 °C for 48 h. Hemolytic patterns were classified as β-hemolysis (clear zones), α-hemolysis (greenish zones), or γ-hemolysis (no zone). Strains showing γ-hemolysis were considered non-hemolytic [16].

### 2.4. Bile Salt Hydrolase (BSH) Activity Assay and Protein Quantification

NES-428 was cultured overnight in MRS broth, and total protein was extracted from the resulting cell pellets using a lysis buffer containing 50 mM Tris-HCl (pH 7.5), 150 mM NaCl, 1% Triton X-100, 1 mM EDTA, and a protease inhibitor cocktail. Protein concentrations were quantified following the manufacturer’s protocol using the BCA Protein Assay Kit (Thermo Fisher Scientific, Waltham, MA, USA), with bovine serum albumin (BSA) as the calibration standard. BSH activity was then evaluated based on a modified protocol from Tanaka et al. [17]. A rapid colorimetric assay was performed in a 96-well microplate by adding 20 μL of crude cell extract to 200 μL of reaction buffer composed of 50 mM sodium phosphate (pH 5.5), 10 mM dithiothreitol (DTT), 1 mM EDTA, and 10 mM sodium glycodeoxycholate. To minimize evaporation and maintain semi-anaerobic conditions during incubation at 37 °C, 50 μL of sterile light paraffin oil was layered onto the reaction mixture. Enzymatic activity was assessed by monitoring the precipitation of deoxycholic acid using a microplate reader. For quantitative determination of BSH activity, a two-step amino acid release assay was conducted. The reaction mixture contained 180 μL of 0.1 M sodium phosphate buffer (pH 6.0), 10 μL of enzyme extract, and 10 μL of a 200 mM mixed human bile salt solution. After incubation at 37 °C for 10 and 30 min, 50 μL aliquots were collected and immediately mixed with 50 μL of 15% (*w*/*v*) trichloroacetic acid to terminate the reaction and precipitate proteins. After centrifugation, the supernatant was mixed with ninhydrin reagent, heated for 14 min, and then cooled. Absorbance was measured at 570 nm. BSH activity was calculated using a glycine standard curve. The glycine concentration in the standard curve was increased to quantify the enzyme activity more accurately.

### 2.5. Plasmid Construction and Adenoviral Vector Packaging

To construct the TSHR289-expressing vector, the backbone plasmid pAV[Exp]-CMV/EGFP, based on the Ad5 gene expression system, was used as an empty control vector. A DNA fragment encoding amino acids 1–289 of the human TSH receptor, which includes nearly the entire A-subunit, was inserted into this vector to generate pAV[Exp]-EGFP-CMV/{TSHR289}, the full sequence of which is available in Supplementary Data 1 [18]. Both vector construction and adenoviral packaging were performed by VectorBuilder (USA) using their custom adenovirus production service.

### 2.6. Constructions of Immunized Mice of Adenovirus Containing TSHR Amino Acid Residues (Ad-TSHR289)

Experimental hyperthyroidism resembling a Graves’ disease mouse model was induced in mice by immunization with a recombinant adenovirus expressing the human thyroid-stimulating hormone receptor (TSHR) A-subunit (Ad-TSHR289). Briefly, female BALB/c mice (age 6 weeks; DooYeol Biotech, Republic of Korea) were injected intramuscularly into hind limbs with 1 × 10^11^ plaque-forming units (PFU) of Ad-TSHR suspended in 50 μL sterile PBS [19]. Mice were monitored weekly for clinical signs, including body weight, general activity, and coat condition, and blood samples were collected at intervals from the tail vein to measure serum thyroxine (T_4_) level using ELISA. Eight to ten weeks after the initial immunization, mice were sacrificed, and blood was collected for terminal hormone analysis. Thyroid glands were excised, and subjected to histological examination by hematoxylin and eosin staining to evaluate follicular morphology. Mice injected with control adenovirus (Ad-LacZ) served as negative controls, ensuring that hyperthyroidism was specifically attributable to TSHR immunization.

### 2.7. Measurement of Serum Thyroxine Levels

Total serum thyroxine (T4) levels were quantified using an enzyme immunoassay kit (Arbor Assays, Ann Arbor, MI, USA) according to the manufacturer’s instructions. Briefly, 10 μL of standards and diluted samples (1:800 in assay buffer) were added to each well, while 35 μL of assay buffer was added to the non-specific binding (NSB) well. Subsequently, 25 μL of conjugate solution was added to all wells, followed by 25 μL of antibody solution to all wells except the NSB. Plates were incubated for 1 h at room temperature with gentle shaking, washed four times with 250 μL wash buffer, and blotted dry. TMB substrate (100 μL) was then added to each well and incubated for 10 min, after which 50 μL of stop solution was added. Absorbance was measured at 450 nm using a SpectraMax plate reader, and T4 concentrations were calculated by a four-parameter logistic (4-PLC) curve fit. Undiluted serum samples were used for all measurements. The intra-assay and inter-assay coefficients of variation (CV) were 3.0% and 6.3%, respectively, as provided by the manufacturer.

### 2.8. Jurkat Cell Culture and Heat-Killed Bacteria Treatment

To investigate the immunomodulatory effects of NES-428, the human T lymphocyte cell line Jurkat (ATCC TIB-152) was employed for cytokine expression analysis. The Jurkat T cell line used in this study was obtained from the Korean Cell Line Bank (KCLB, Seoul, Republic of Korea). Jurkat T cells were cultured at 37 °C in a humidified incubator with 5% CO_2_ using RPMI 1640 medium (Gibco) supplemented with 10% heat-inactivated fetal bovine serum (FBS), 100 µg/mL penicillin, and 100 U/mL streptomycin. For recovery from cryopreservation, frozen vials (~1 mL) were rapidly thawed in a 37 °C water bath for 1–2 min until only a small ice crystal remained, then transferred to a biosafety cabinet and diluted with 4 mL of pre-warmed medium (final volume 5 mL). The suspension was centrifuged at 1500 rpm for 10 min, the supernatant containing DMSO was discarded, and cells were resuspended in 5 mL fresh medium before seeding into a 60 mm dish. After 24 h, cells were collected, washed, and resuspended in 10 mL of fresh medium and transferred to a 100 mm dish. For maintenance, cells were passaged every 2–3 days by centrifugation at 1500 rpm for 10 min, removal of spent medium, and resuspension in fresh medium, with seeding at a density of 1.5 × 10^6^ cells/10 mL per 100 mm dish and supplementation with an additional 5 mL of fresh medium after 2 days. NES-428 cultures were heat-killed by incubation at 75 °C for 30 s, and successful inactivation was confirmed by the absence of growth on MRS agar plates. Jurkat cells were seeded at a density of 1 × 10^6^ cells/mL in RPMI 1640 medium supplemented with 10% fetal bovine serum (FBS) and antibiotics [20]. Heat-killed NES-428 was added to the cells at a defined multiplicity (e.g., 10^8^ CFU-equivalent/mL) and incubated for 24 h at 37 °C in a 5% CO_2_ atmosphere. The heat-killed NES-428 strain was prepared by incubation at 80 °C for 30 min, after which the wet weight of the bacteria was measured, and an equivalent amount corresponding to 1 × 10^8^ cells/mL was applied to Jurkat cells. We used 5 × 10^6^ Jurkat cells for each experimental condition. In the non-treated (NC) group, the cells were simply maintained for 24 h without any stimulation. In the stimulated control (ST) group, the same number of cells was exposed to 50 ng/mL of phorbol 12-myristate 13-acetate (PMA) and 1 µg/mL ionomycin for 6 h. For the NES-428 group, Jurkat cells were first incubated for 24 h with heat-killed NES-428, after which they are stimulated by 50 ng/mL PMA and 1 µg/mL ionomycin for 6 h. Total RNA was extracted from each group, and cytokine expression was analyzed by quantitative real-time PCR.

### 2.9. RNA Extraction from Jurkat Cell and Spleen Tissue

Total RNA was extracted from jurkat cells and spleen tissues using the RNeasy Mini Kit (Qiagen, Germantown, MD, USA) following the manufacturer’s instructions. Briefly, samples were homogenized in buffer RLT, and RNA was isolated by column purification. The integrity and quantity of the RNA were assessed using a NanoDrop spectrophotometer (Thermo Fisher Scientific, Waltham, MA, USA) and by agarose gel electrophoresis

### 2.10. mRNA Expression Analysis by qRT-PCR

cDNA was synthesized using a reverse transcription kit (iScript cDNA Synthesis Kit, Bio-Rad, Hercules, CA, USA), and quantitative real-time PCR (RT-qPCR) was conducted using SYBR Green Master Mix (Thermo Fisher Scientific, Waltham, MA, USA) on a real-time PCR system. Gene-specific primers were used for IL-2, IL-4, IL-6, IL-12, TNF-α, and IFN-γ, with GAPDH serving as the internal control. Relative gene expression levels were calculated using the ΔΔCt method. The primer sequences are provided in Table 2.

### 2.11. Histopathological Analysis of Thyroid Tissue

To examine anatomical changes in the thyroid tissue, histopathological analysis was performed. Thyroid samples from each group were collected, fixed in 10% formalin, and processed for paraffin embedding. Tissue sections of 5 μm thickness were prepared and stained with hematoxylin and eosin (H&E). Histopathological changes in the thyroid were evaluated by examining follicular morphology, epithelial cell height, colloid appearance, and inflammatory infiltration. Normal control mice showed uniformly sized follicles lined by cuboidal epithelium with colloid-filled lumina. In contrast, AdTSHR-immunized mice exhibited epithelial hypertrophy and hyperplasia with columnar cells, colloid depletion with scalloping, irregularly sized follicles, and occasional lymphocytic infiltration, which are typical features of hyperthyroidism. In the AdTSHR/NES-428 group, these pathological changes were attenuated, with relatively preserved follicular architecture, reduced epithelial thickening, and improved colloid retention compared to the AdTSHR group.

### 2.12. Ethics Approval

All animal procedures were approved by the Institutional Animal Care and Use Committee (IACUC) of Korea University (Approval No. KUIACUC-2023-0023, approved on 11 April 2023), and were humanely euthanized using CO_2_ gas chamber according to the IACUC guidelines, Korea University. Additionally, this study was conducted in accordance with institutional guidelines and the principles of the Declaration of Helsinki (revised in 2013).

## 3. Results

### 3.1. Construction of Hyperthyroidsm Mouse Model

**Figure 1:**
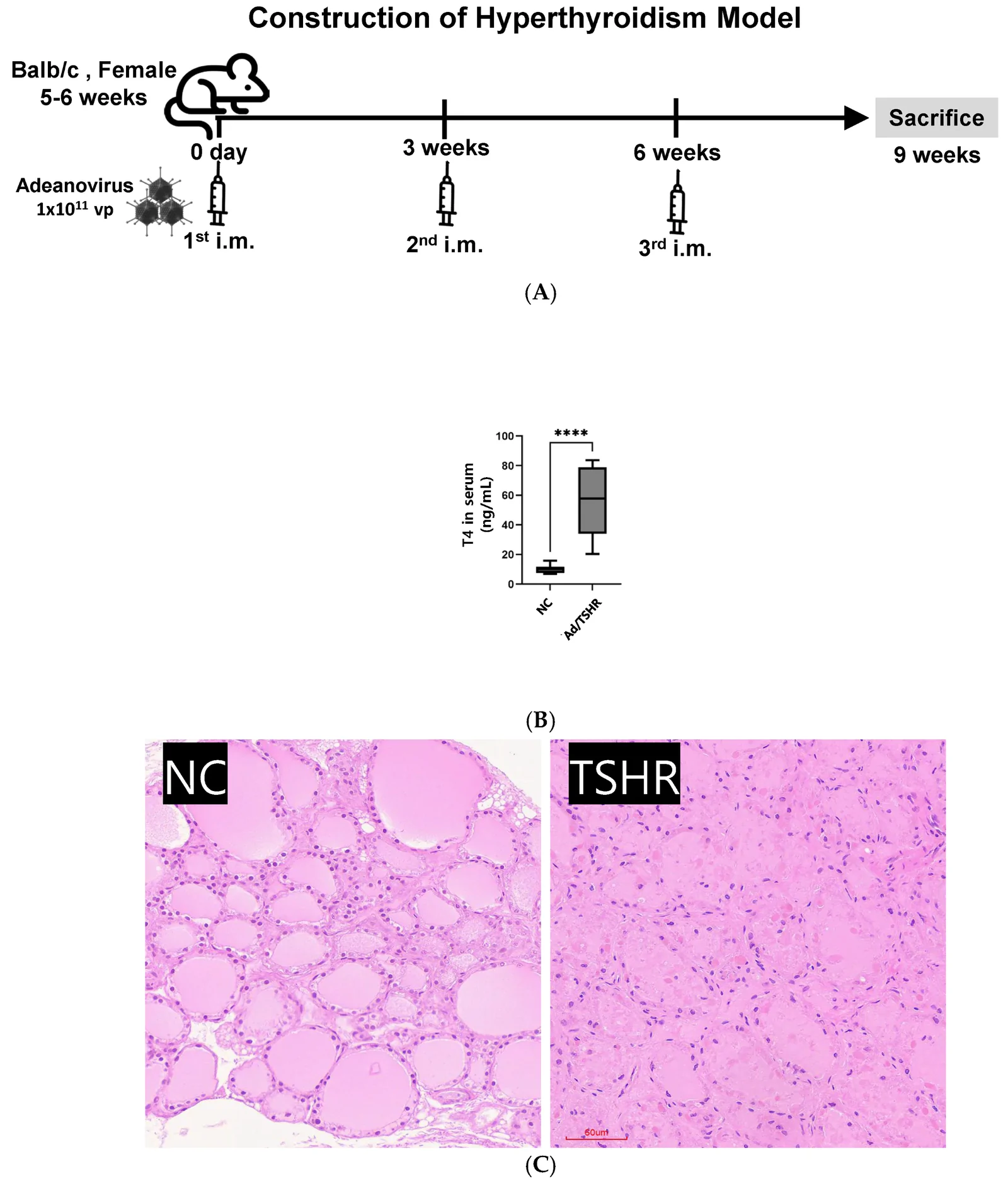
Construction of the experimental hyperthyroidism model. (**A**) Timeline for adenovirus-TSHR immunization and intramuscular (i.m.) boosts in female BALB/c mice (5-6 weeks old). Schematic of three sequential i.m. injections with Ad-TSHR (1 × 10^11^ vp) given at weeks 0, 3 and 6. (**B**) Serum thyroxine (T_4_) concentration in non-treated (NC) versus TSHR-immunized (TSHR) mice at week 9; each point represents an individual animal (mean ± SD; **** *p* < 0.0001 vs. NC, one-way ANOVA). (**C**) Representative haematoxylin-and-eosin sections of thyroid tissue: NC (healthy control) and TSHR (hyperthyroid). TSHR mice markedly increase follicular hyperplasia and colloid depletion compared with NC mice (scale bar = 50 µm).

To generate a murine model of hyper-thyroidism, the extracellular A-subunit of the human TSH receptor (TSHR; amino acid residues 1–289) was amplified by PCR from human TSHR cDNA and sub-cloned into an adenoviral vector (Ad/TSHR) constructed by VectorBuilder (Chicago, IL, USA). This ectodomain harbors the principal immunogenic epitopes that elicit thyroid-stimulating autoantibodies (TSAbs) [19]. As outlined in Figure 1A, female BALB/c mice were immunized intramuscularly with 1 × 10^11^ viral particles of Ad/TSHR on days 0, 21, and 42. A negative-control group received the empty adenoviral vector (NC, *n* = 5). Serum was collected three weeks after the final injection. Relative to controls, Ad/TSHR-immunized mice displayed a marked increase in total thyroxine (T_4_) concentrations (Figure 1B; *p* < 0.01), confirming successful induction of a hyperthyroid state. Histological examination of thyroid sections revealed disrupted follicular architecture and irregular epithelial hypertrophy in Ad/TSHR mice (Figure 1C), further validating the establishment of the model.

### 3.2. Isolation and Identification of Lactic Acid Bacteria from Kimchi

**Figure 2:**
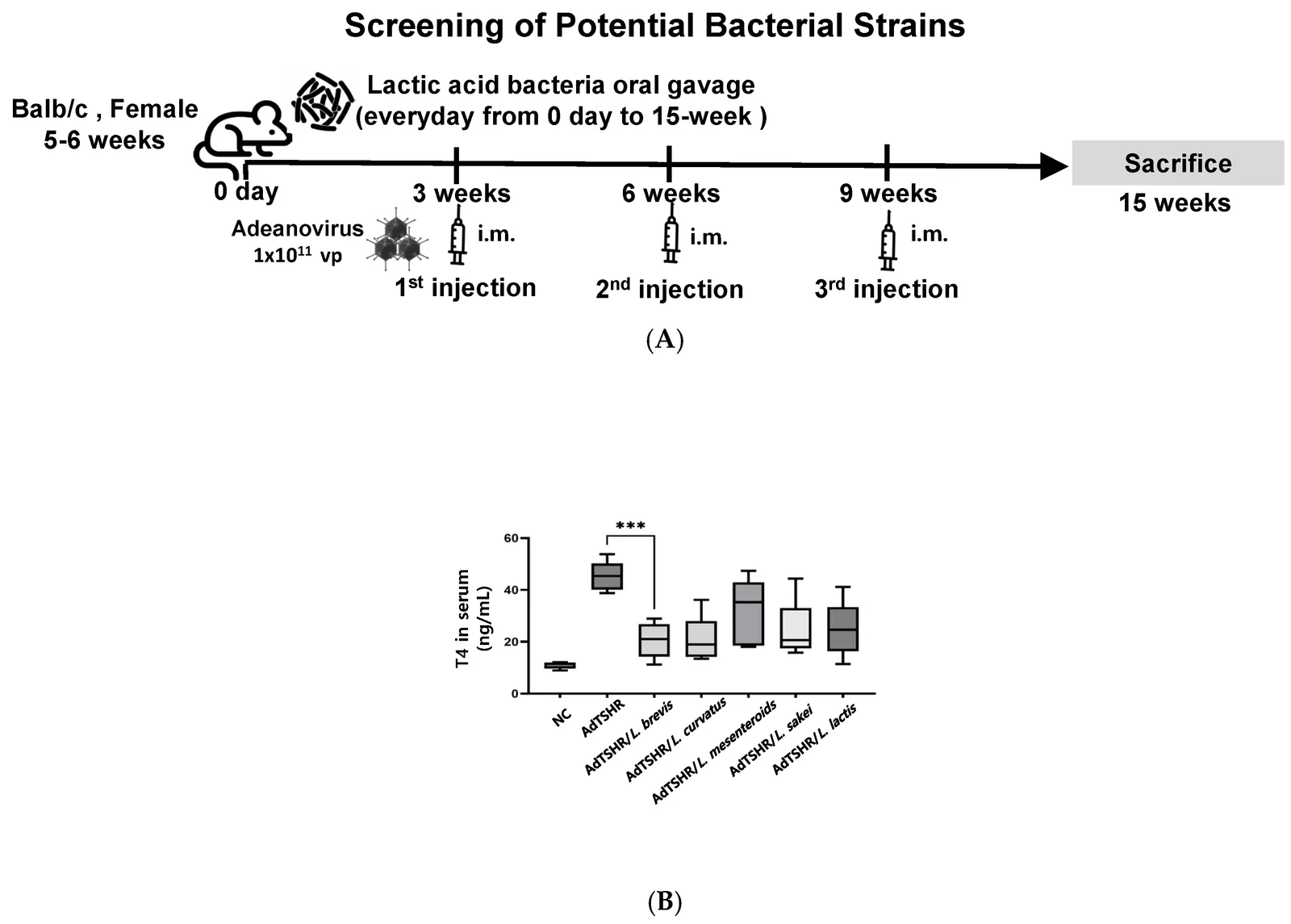
Preliminary screening of lactic acid bacterial (LAB) strains for anti-hyperthyroid activity. (**A**) Experimental schedule combining three i.m. Ad-TSHR injections with daily oral gavage of candidate LAB isolates from day 0 to week 15. (**B**) Serum T_4_ levels at week 15 after treatment with the indicated LAB strains; dashed line = TSHR group mean. Bars show mean ± SD (*n* = 5). Asterisks denote significance vs. TSHR-only (*p* < 0.05; *** *p* < 0.01).

**Figure 3:**
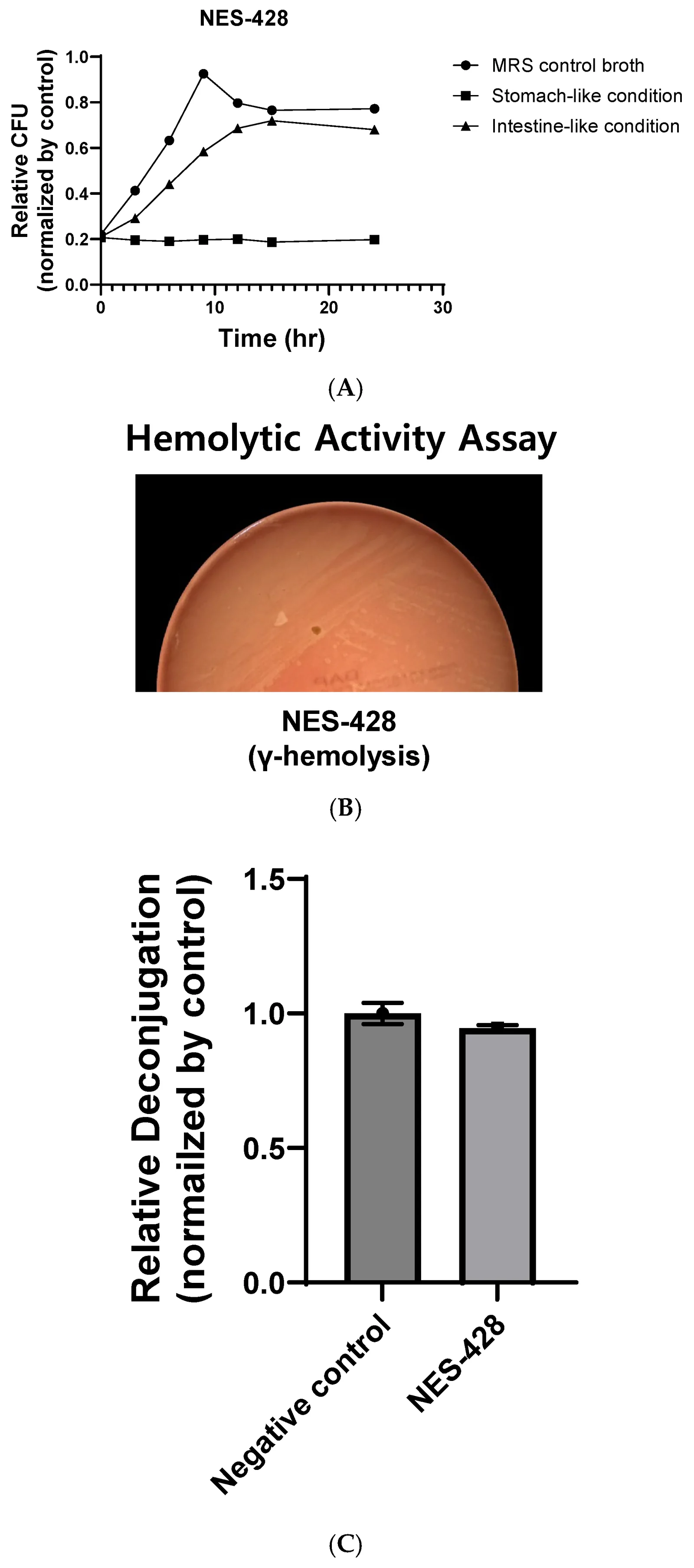
Gastro-intestinal survivability and safety profile of strain NES-428. (**A**) Relative CFU of NES-428 in simulated stomach (pH 2.5, pepsin) and intestine (pH 7.4, bile salts) media over 30 h, normalized to MRS control broth. (**B**) γ-haemolysis on sheep-blood agar demonstrating absence of haemolytic activity (NES-428). (**C**) Bile-salt-hydrolase (BSH) deconjugation activity expressed as relative OD_600_; NES-428 shows minimal BSH compared with positive control (mean ± SD, *n* = 3). As a negative control, the vehicle without any addition was used.

Probiotics are generally known to exert beneficial effects on the human immune system [12,29]. In this study, to identify probiotics effective against Graves’ disease, a type of autoimmune disorder, we isolated lactic acid bacteria from hundreds of different kimchi samples collected from restaurants and households across Korea. We isolated several bacterial strains from kimchi and identified them by 16S rRNA gene sequencing. Phylogenetic analysis showed that each isolate shares ≥ 99% sequence identity with its closest type strain recorded in public databases, confirming that our cultures represent novel strains distinct from those previously described. Among the isolates, five strains that have been previously reported to be beneficial to human health were selected. These five selected strains were *Lactobacillus brevis*, *Lactobacillus curvatus*, *Lactobacillus lactis*, *Lactobacillus sakei*, and *Leuconostoc mesenteroides* [30,31,32,33]. They were then evaluated for their potential preventive effects against Graves’ disease using the hyperthyroidism animal model established as shown in Figure 1. Five strains were pretreated to mice orally for 3 weeks before TSHR immunization and TSHR was immunized by three injections at 3-week intervals, and serum T4 levels were measured as shown in Figure 2A. As shown in Figure 2B, mice administered with the five selected probiotic strains tended to lower serum T4 levels compared to untreated mice. Among these, *L. brevis* showed the most pronounced reduction in T4 levels and was named NES-428. Based on these results, NES-428 was selected for further investigation in this study. NES-428 showed 99% 16S-rRNA sequence identity to previously described Lactobacillus brevis isolates, indicating that it represents a previously unreported strain of this species (Figure S1). To assess the safety and probiotic characteristics of NES-428, several in vitro assays were conducted. Figure 3A shows the viability of NES-428 in simulated gastric juice (pH 2.5) and bile salts. NES-428 maintained viability throughout the 3 h exposure period. And NES-428 was able to grow in media supplemented with 0.3% bile salts (Figure 3A). Furthermore, NES-428 did not exhibit any hemolytic activity on blood agar plates, and it also tested negative for bile salt deconjugation (Figure 3B,C).

### 3.3. NES-428 Has an Immunomodulatory Function

**Figure 4:**
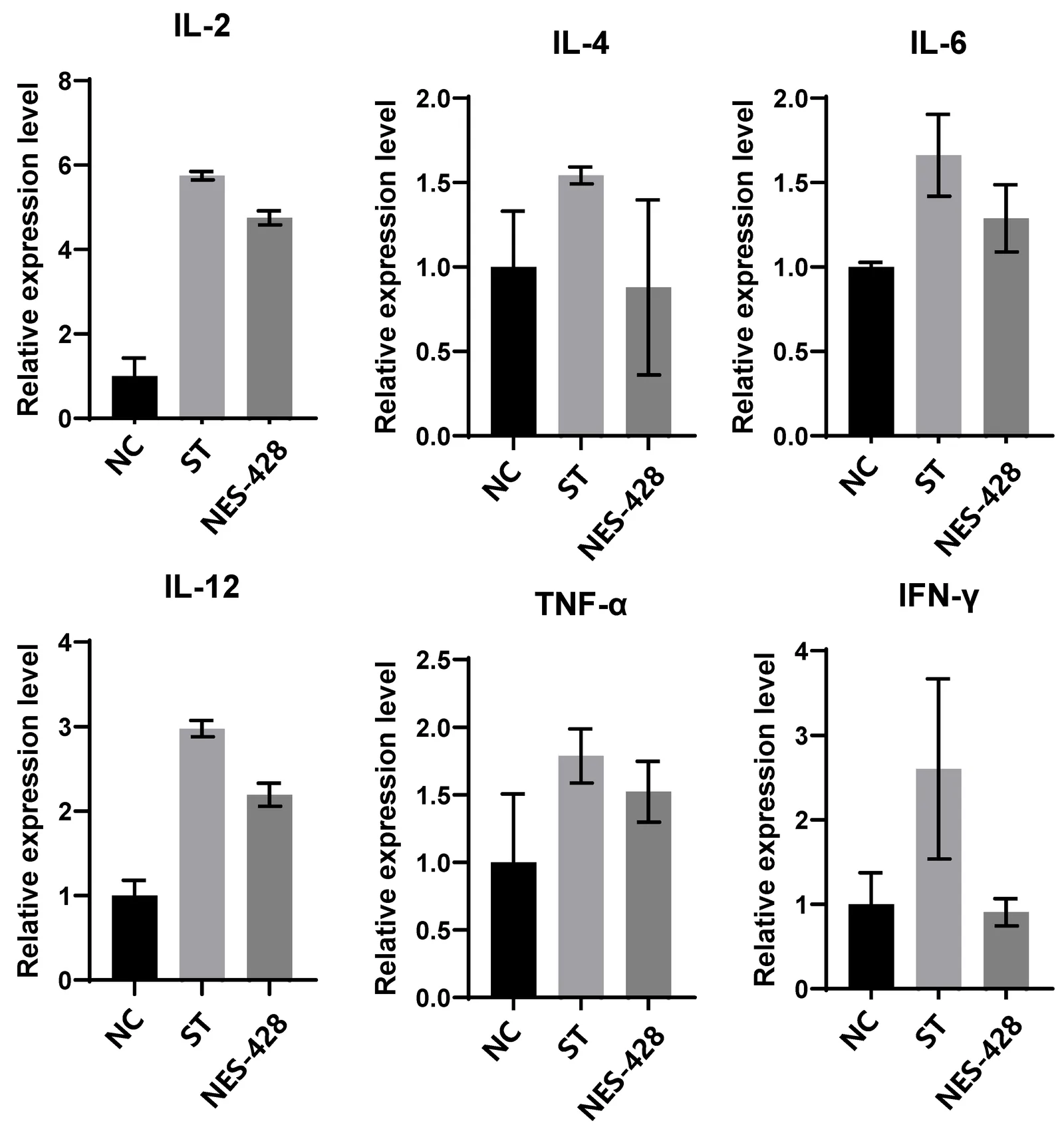
NES-428 modulates pro- and anti-inflammatory cytokine transcription in Jurkat T cells. In the non-treated (NC) group, the cells were simply maintained for 24 h without any stimulation. In the stimulated control (ST) group, the same number of cells was exposed to 50 ng/mL of phorbol 12-myristate 13-acetate (PMA) and 1 µg/mL ionomycin for 6 h. For the NES-428 group, Jurkat cells were first incubated for 24 h with heat-killed NES-428, after which they were stimulated by 50 ng/mL PMA and 1 µg/mL ionomycin for 6 h. Total RNA was extracted from each group, and cytokine expression was analyzed by quantitative real-time PCR. Relative mRNA expression of IL-2, IL-4, IL-6, IL-12, TNF-α and IFN-γ was shown. NC = untreated control; ST = stimulated positive control. Data represent mean ± SD of three independent experiments; *p* < 0.05 vs. NC.

To evaluate the immunomodulatory effects of NES-428, we first examined cytokine expression changes using the human T cell line, Jurkat cells. NES-428 was heat-killed before treatment and then applied to Jurkat cells, followed by assessment of cytokine expression levels using RT-qPCR as described in materials and methods [34]. As shown in Figure 4, the expression levels of IL-2, IL-4, IL-6, IL-12, and TNF-α were reduced, whereas IFN-γ expression remained unchanged [20]. These results suggest that NES-428 may exert immunomodulatory effects by selectively downregulating pro-inflammatory and T cell-associated cytokines such as IL-2, IL-4, IL-6, IL-12, and TNF-α in jurkat cells. The lack of change in IFN-γ expression indicates that NES-428 may not significantly impact Th1-type immune responses but rather modulates broader inflammatory signaling pathways [12,35]. This selective cytokine modulation implies that NES-428 could help regulate immune activation without broadly suppressing cellular immunity, making it a promising candidate for the management of autoimmune diseases such as Graves’ disease.

**Figure 5:**
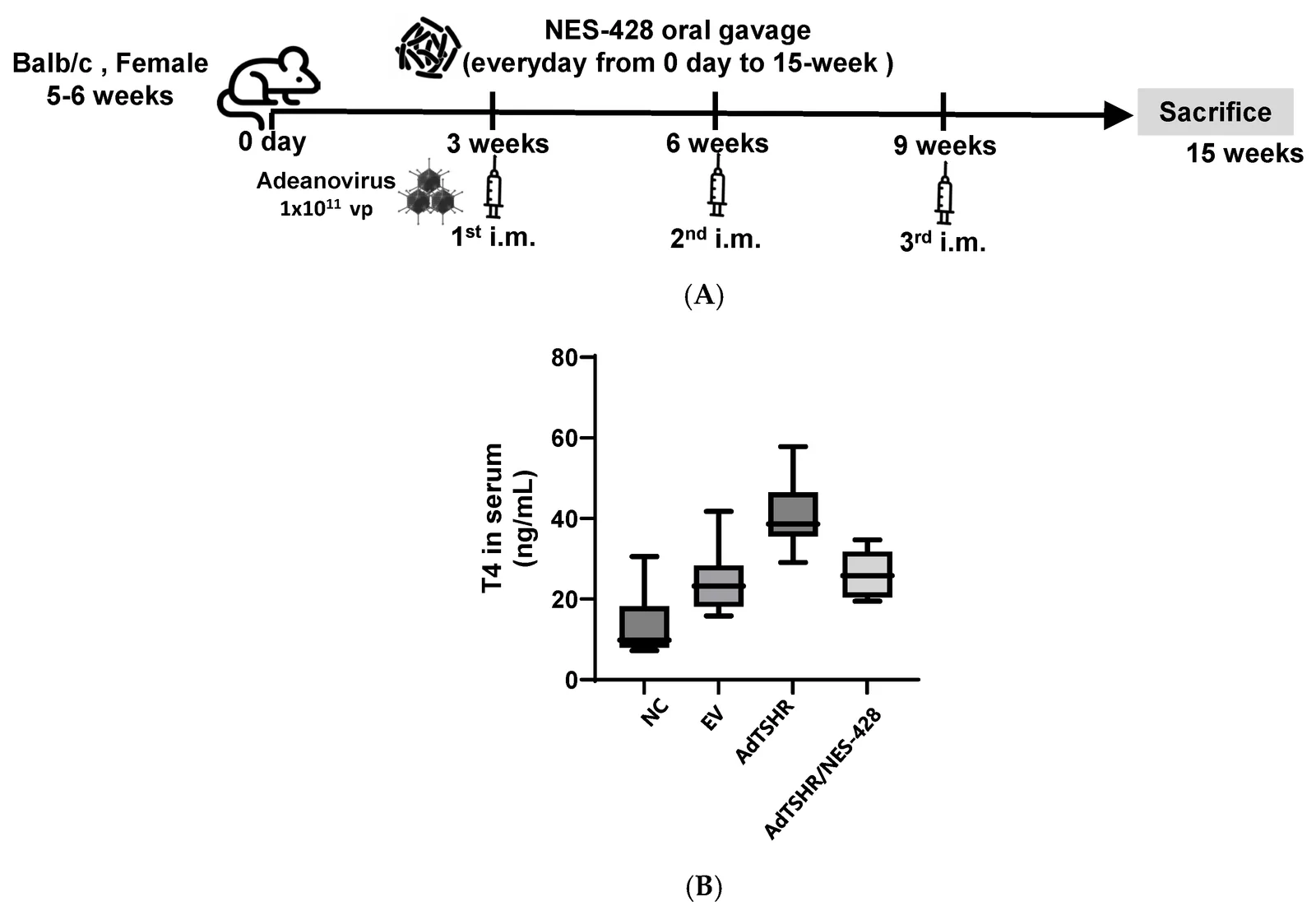
Long-term suppression of hyperthyroidism by daily oral NES-428 in Ad-TSHR mice. (**A**) Study timeline identical to Figure 2 but using the single best-performing strain NES-428. (**B**) Serum T_4_ at week 15 for NC, Ad-TSHR only (TSHR), and Ad-TSHR + NES-428 groups (*n* = 6). NES-428 significantly lowers T_4_ compared with TSHR alone.

We next evaluated the in vivo efficacy of NES-428 in the hyperthyroid mouse model. As depicted in Figure 5, NES-428 supplementation markedly blunted the rise in serum thyroxine (T_4_) triggered by Ad/TSHR immunization. In the TSHR group, mean T_4_ concentrations climbed steadily during the 15-week observation period, reaching 38 pg/mL at week 15 (*p* < 0.05 vs. NC). By contrast, mice receiving NES-428 maintained significantly lower T_4_ values throughout the study; at week 15 the TSHR + NES-428 group averaged 25 pg/mL, representing a 34% reduction relative to TSHR mice (*p* < 0.05). These results demonstrate that oral NES-428 effectively mitigates the hyperthyroid phenotype induced by TSHR overexpression in vivo.

**Figure 6:**
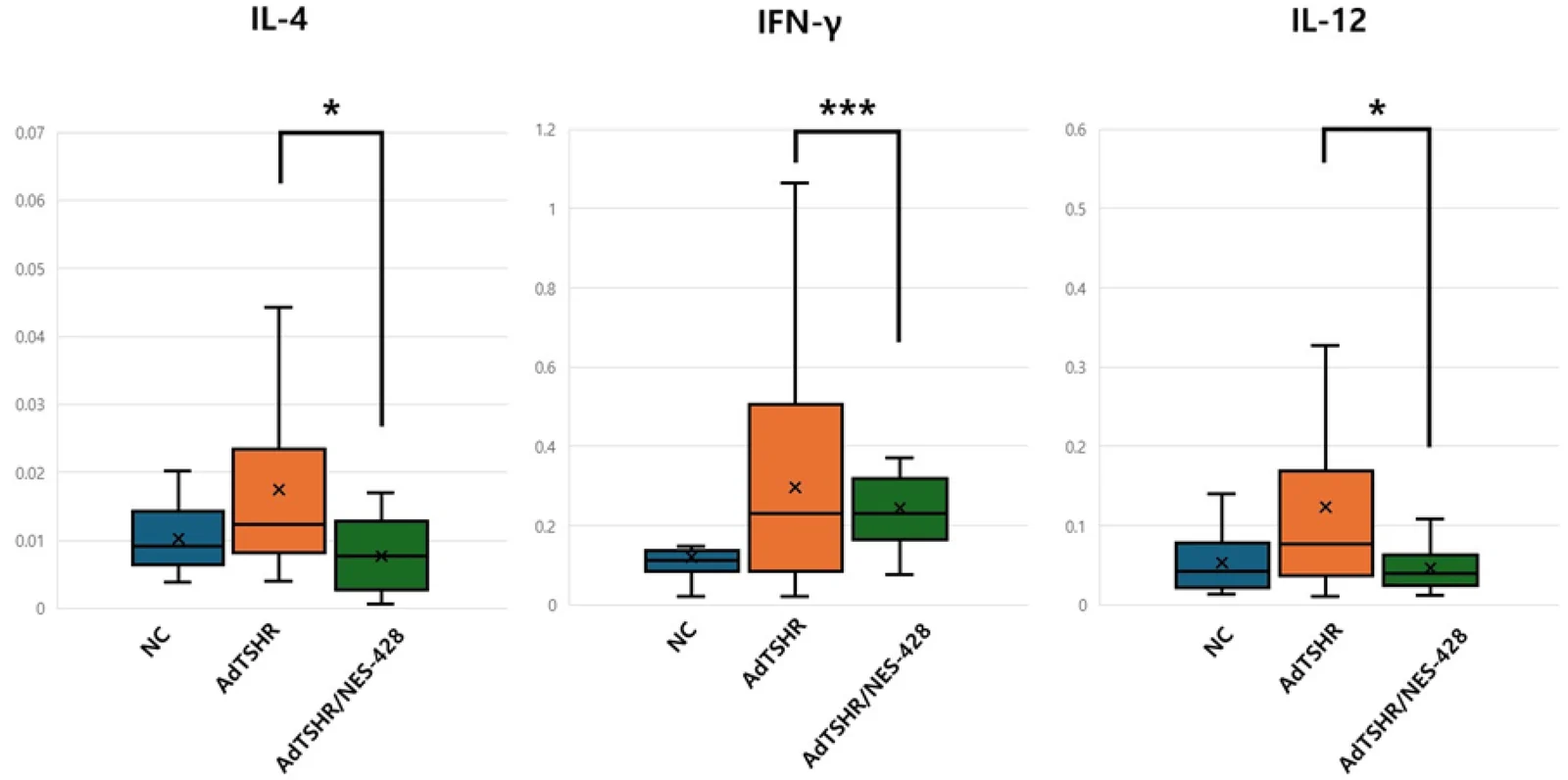
NES-428 shifts splenocyte cytokine production toward a Th1-suppressive profile in Ad-TSHR mice. Splenocytes were harvested on day 105 (week 15) from non-treated controls (NC), Ad-TSHR-immunized hyperthyroid mice (TSHR), and Ad-TSHR mice that had received daily oral NES-428 (1 × 10^9^ CFU) for the entire study period (TSHR + NES-428). Cells (2 × 10^6^/well) were re-stimulated ex vivo for 48 h with recombinant TSHR A-subunit (5 µg/mL). Culture supernatants were analyzed by ELISA for IFN-γ, IL-12p70, and IL-4. Bars represent mean ± SD (n = 6 mice per group, assays in duplicate). * *p <* 0.05 versus TSHR group, and *** *p <* 0.001 versus TSHR group (one-way ANOVA followed by Tukey’s post hoc test).

The immunomodulatory effects of NES-428 were further investigated by analyzing the expression levels of key cytokines involved in Graves’ disease pathogenesis. Spleenic RNA extracts were used to measure the mRNA expression levels of IL-4 (Th2-related), IL-12 (Th1-related), and IFN-γ (Th1-related) via RT-qPCR. As shown in Figure 6, the TSHR group exhibited elevated levels of all three cytokines compared to the normal control group, indicating immune dysregulation typical of hyperthyroidism. However, NES-428 supplementation significantly modulated these cytokine levels. Specifically, IL-4 expression was reduced from ~0.06 relative units in the TSHR group to ~0.02 relative units in the TSHR/NES-428 group (*p* < 0.05). Similarly, IL-12 expression decreased from ~0.50 to ~0.20 relative units (*p* < 0.01), and IFN-γ expression was reduced from ~0.07 to ~0.03 relative units (*p* < 0.05). These results demonstrate that NES-428 can modulate both Th1 and Th2 immune responses in the hyperthyroidism mouse model [36].

**Figure 7:**
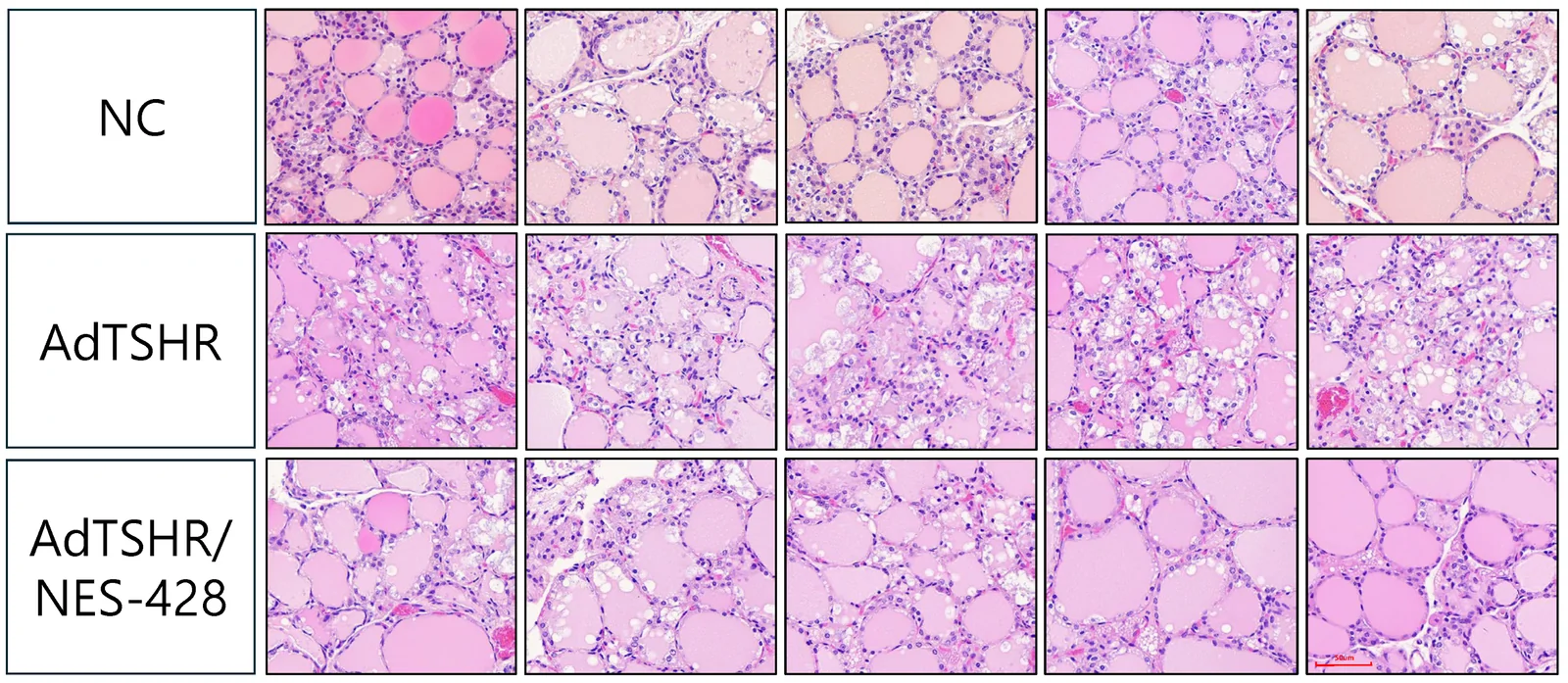
NES-428 normalizes thyroid histopathology in hyperthyroid mice. Representative hematoxylin-and-eosin sections of thyroid tissue: NC (healthy control), TSHR (hyperthyroid), and TSHR + NES-428. NES-428 markedly reduces follicular hyperplasia and colloid depletion compared with TSHR mice (scale bar = 50 µm).

Histopathological analysis was performed to assess the extent of thyroid inflammation in each group. Representative thyroid sections stained with H&E are shown in Figure 7. The normal control (NC) group exhibited normal thyroid follicular architecture with minimal inflammatory infiltration. In contrast, the TSHR-induced hyperthyroidism group showed significant thyroid inflammation characterized by increased lymphocytic infiltration, follicular disruption, and signs of thyrocyte hypertrophy [37]. Notably, the TSHR/NES-428 group exhibited reduced inflammatory cell infiltration and less disruption of the thyroid tissue structure, with a more preserved follicular architecture compared to the TSHR group [38,39,40,41]. These findings support the conclusion that NES-428 can reduce thyroid inflammation in the hyperthyroidism mouse model.

## 4. Discussion

The present study demonstrates that oral administration of NES-428 significantly ameliorates hyperthyroidism induced by TSHR overexpression in a murine hyperthyroidism model, supporting the potential of NES-428 as a preventive agent for autoimmune thyroid disorders such as Graves’ disease. The key findings include a reduction in serum T4 levels, modulation of Th1/Th2 cytokine responses, and attenuation of thyroid inflammation. These observations are consistent with the hypothesis that NES-428 can restore immune homeostasis in the context of GD by targeting key pathogenic mechanisms.

The observed reduction in serum T4 levels in the TSHR/NES-428 group suggests that NES-428 can directly or indirectly interfere with the TSHR signaling pathway and/or thyroid hormone synthesis [42]. Although the precise mechanisms remain to be fully elucidated, the concurrent modulation of cytokine profiles provides valuable insights [43]. As shown in our Jurkat T-cell model experiment, heat-killed NES-428 treatment led to a decrease in IL-2, IL-4, IL-6, IL-12, and TNF-α. In the animal model study described herein, the significant reduction in the levels of IL-4, IL-12, and IFN-γ in the TSHR/NES-428 group, particularly from the cytokine expression data, underscores the immunomodulatory potential of NES-428. This dual modulation of Th1/Th2 pathways suggests that NES-428 could serve as a preventive agent for GD by restoring immune homeostasis. These patterns can be interpreted as the following. First, there was a reduction in IL-4, a key cytokine involved in B-cell activation and the production of autoantibodies, including TRAb, in GD.6 [44]. By suppressing IL-4 expression, NES-428 may directly inhibit TRAb synthesis, thereby reducing the stimulatory effect on thyroid follicular cells and lowering T4 production [45,46]. Second, by downregulating IL-12 and IFN-γ expression, NES-428 may attenuate the inflammatory cascade in the thyroid gland, preventing further tissue destruction [47]. As previously shown in animal models of IBD where suppression of TNF-α was observed, modulating cytokine profiles led to a reduction in disease severity [48]. Finally, balanced regulation of IFN-γ may allow NES-428 to reduce the immune responses without diminishing base levels of immunity, which is a distinct advantage over other treatment [49,50].

As the core of the autoimmune process in Graves’ disease lies in TRAb production and Th1/Th2 overactivation, these results indicate that probiotics have sufficient potential not only as simple modulators but also as candidates for preventive agents [51]. The findings from this study are in line with the hypothesis that *Lactobacillus* strains modulate the host immune system through various mechanisms, including direct interaction with immune cells, alteration of gut microbiota composition, and production of immunomodulatory metabolites Our results showed that heat-killed *Lactobacillus* induced down-regulation of IL-2, IL-4, IL-6, IL-12 and TNF-α, coupled with IFN-γ regulation in Jurkat T cells, which is particularly noteworthy [52,53,54]. We believe that in Jurkat T cells, *Lactobacillus* induces a more complex immunomodulatory behavior that must be studied further for its clinical applications. However, what can be said with relative certainty is that these observations suggest NES-428 may play a key role in maintaining overall immunity and preventing thyroid dysfunction.

The histopathological analysis further supports the immunomodulatory effects of NES-428. The reduced lymphocytic infiltration and preserved thyroid architecture observed in the TSHR/NES-428 group indicate that NES-428 can mitigate the inflammatory response in the thyroid gland, preventing extensive tissue damage. These structural improvements are closely related to the reduction in thyroid T4 levels that are seen. While this study provides compelling evidence for the immunomodulatory potential of NES-428 in a hyperthyroidism mouse model, several limitations need to be addressed in future research. First, although we observed significant modulation of cytokine profiles, the precise mechanisms by which NES-428 exerts its effects remain unclear. Future studies should investigate the direct interactions of NES-428 with immune cells, its impact on gut microbiota composition, and the role of immunomodulatory metabolites produced by NES-428 [55]. In short, further mechanistic analyses are required to characterize these effects. Second, regulatory T cells (Tregs) play a critical role in maintaining immune homeostasis and preventing autoimmune diseases. Although we believe that there is an induction, additional analyses, including the evaluation of Treg markers (FoxP3, IL-10, TGF-β), are required to confirm this [8,56]. Third, the TSHR-induced hyperthyroidism mouse model, while useful for studying GD, does not fully replicate the complexity of human GD [40,57]. Further studies using more sophisticated animal models that mimic the genetic and environmental factors involved in GD pathogenesis are warranted [58]. And finally, this study primarily relies on a hyperthyroidism-induced animal model. Clinical trials in humans, with analysis of disease progression metrics, are required.

## 5. Conclusions

This study identifies the kimchi-derived strain NES-428 as having anti-thyroid activity. In vitro, NES-428 tempered Th1-skewed cytokine expression in Jurkat T cells, suggesting a direct T-cell regulatory effect. In vivo, daily oral administration of NES-428 significantly lowered serum thyroxine concentrations, preserved normal follicular architecture, and shifted splenocyte cytokine output toward a Th2 profile in an Ad/TSHR-induced hyperthyroid mouse model. These findings demonstrate that NES-428 can attenuate the autoimmune hyperthyroid phenotype by dampening pathogenic cell-mediated responses and restoring hormonal balance. Although the mechanism appears to involve suppression of IFN–γ–dominated signaling, additional work is required to map the gut–thyroid axis and to determine whether live or post-biotic components mediate the effect. Taken together, NES-428 represents a promising probiotic candidate for adjunct management of Graves’ disease and other thyroid-mediated autoimmune disorders.

## Figures and Tables

**Table 1:** Primer List for 16S rRNA sequencing.

Primer Name	Sequence (5′ → 3′)
27F	AGAGTTTGATCMTGGCTCAG
337F	ACTCCTACGGGAGGCAGCAG
518F	CCAGCAGCCGCGGTAATAC
785F	GGATTAGATACCCTGGTA
518R	ATTACCGCGGCTGCTGG
783R	ACAGGGTATCTAATCCTGTT
805R	GACTACCAGGGTATCTAATCC
907R	CCGTCAATTCMTTTRAGTTT
1492R	GGTTACCTTGTTACGACTT

**Table 2:** Primer List for qRT-PCR.

Gene	Sequence(5′ → 3′)	Ref.
human IL-2	AAGAATCCCAAACTAACCAGGAT	[21]
	TCTAGACATGAAGATGTTTCAGTTCTC	
mouse IL-2	TGATGGACCTACAGGAGCTCCTGAG	[22]
	GAGTCAAATCCAGAACATGCCGCAG	
hIL-4	AGATCATCGGCATTTTGAAC	[23]
	TTTGGCACATCCATCTCCG	
mIL-4	ATCATCGGCATTTTGAACGAGGTC	[24]
	ACCTTGGAAGCCCTACAGACGA	
hIL-6	GTCAACTCCATCTGCCCTTCAG	[25]
	GGTCTGTTGTGGGTGGTATCCT	
mIL-6	CCCCAATTTCCAATGCTCTCC	[22]
	CGCACTAGGTTTGCCGAGTA	
hIL-12	GACATTCTGCGTTCAGGTCCAG	[26]
	CATTTTTGCGGCAGATGACCGTG	
mIL-12	AAATGAAGCTCTGCATCCTGC	[27]
	TCACCCTGTTGATGGTCACG	
hTNF-α	CCGAGGCAGTCAGATCATCTT	[21]
	AGCTGCCCCTCAGCTTGA	
mTNF-α	ACCCTCACACTCACAAACCA	[22]
	ATAGCAAATCGGCTGACGGT	
hIFN-γ	TGTAGCGGATAATGGAACTCTTTT	[21]
	AATTTGGCTCTGCATTATT	
mIFN-γ	GAACTGGCAAAAGGATGGTGA	[22]
	TGTGGGTTGTTGACCTCAAAC	
hGAPDH	AGGTTGTCTCCTGCGACT	[28]
	TGCTGTAGCCGTATTCATTGTCA	
mGAPDH	AAATGGTGAAGGTCGGTGTG	[22]
	TGAAGGGGTCGTTGATGG	

## Data Availability

The original contributions presented in this study are included in the article/Supplementary Material. Further inquiries can be directed to the corresponding author.

## Supplementary Materials

The following supporting information can be downloaded at https://www.mdpi.com/article/10.3390/nu17182967/s1, Figure S1: The nucleotide sequence of 16S ribosomal DNA of NES-428 Sequencing analysis showed that the kimchi-derived strain NES-428 shares approximately 99% nucleotide identity with previously characterized Lactobacillus brevis strains.
