# Supplementary material for: Immunomodulatory Effects of Lactobacillus brevis NES-428 in a Hyperthyroidism Mouse Model: Potential Applications for Graves’ Disease
Source: Nutrients. 2025 Sep 16;17(18):2967. doi: 10.3390/nu17182967 (PMC12473064; doi:10.3390/nu17182967)
Supplement: Supplementary file 1 [file nutrients-17-02967-s001.zip › nutrients-3823825-supplementary.pdf]

Figure S1. The nucleotide sequence of 16S ribosomal DNA of NES-428 Sequencing analysis showed that the kimchi-derived strain NES-428 shares approximately 99 % nucleotide identity with previously characterized *Lactobacillus brevis* strains.

>239366-MA-043-1-Kimchi4-5-518F

GGGGACCGTGCTCGGGATTTATTGGGCGTAAGCGAGCGCAGGCGGTTTTTTAAGTCTGATGTGAAAGCCTTC
GGTTTAACCGGAGAAGTGCATCGGAAACTGGGAGACTTGAGTGCAGAAGAGGACAGTGGAACTCCATGTGT
AGCGGTGGAATGCGTAGATATATGGAAGAACACCAGTGGCGAAGGCGGCTGTCTAGTCTGTAACTGACGCTG
AGGCTCGAAAGCATGGGTAGCGAACAGGATTAGATACCCTGGTAGTCCATGCCGTAAACGATGAGTGCTAAGT
GTTGGAGGGTTTCCGCCCTTCAGTGCTGCAGCTAACGCATTAAGCACTCCGCCTGGGGAGTACGACCGCAAG
GTTGAAACTCAAAGGAATTGACGGGGGCCCGCACAAGCGGTGGAGCATGTGGTTTAATTCGAAGCTACGCG
AAGAACCTTACCAGGTCTTGACATCTTCTGCCAATCTTAGAGATAAGACGTTCCCTTCGGGGACAGAATGACA
GGTGGTGCATGGTTGTCGTCAGCTCGTGTCGTGAGATGTTGGGTTAAGTCCCGCAACGAGCGCAACCCTTATTA
TCAGTTGCCAGCATTCAGTTGGGCACTCTGGTGAGACTGCCGGTGACAAACCGGAGGAAGGTGGGGATGAC
GTCAAATCATCATGCCCCTTATGACCTGGGCTACACACGTGCTACAATGGACGGTACAACGAGTTGCGAAGTC
GTGAGGCTAAGCTAATCTCTTAAAGCCGTTCTCAGTTCGGATTGTAGGCTGCAACTCGCCTACATGAAGTTGGA
ATCGCTAGTAATCGCGGATCAGCATGCCGCGGTGAATACGTTCCCGGGCCTTGTACACACCGCCCGTCACACC
ATGAGAGTTTGTAACACCCAAAGCCGGTGAGATAACCTTCGGGAGTCAGCCGTCTAAGGTGGGACAGATGAT
TAGGGTGAAGTCGTACAAGGGGGAAACCGTAAAA
